# Self-Reported Dietary Carotenoid Intake and Skin Carotenoid Score Status in a Geographically Diverse U.S. Sample

**DOI:** 10.3390/nu18152515

**Published:** 2026-08-03

**Authors:** Saima Hasnin, Susan B. Sisson, Stephanie Jilcott Pitts, Justin D. Dvorak, Taren Massey Swindle, Shanon L. Casperson, Ashlea Braun, Jonathan Baldwin, Emily Lynn, Virginia C. Stage

**Affiliations:** 1 Department of Food Science and Human Nutrition, University of Illinois Urbana-Champaign, Urbana, IL 61801, USA; 2 Department of Nutritional Sciences, University of Oklahoma Health Campus, Oklahoma City, OK 73117, USA; susan-sisson@ou.edu; 3 Department of Public Health, East Carolina University, Greenville, NC 27834, USA; jilcotts@ecu.edu; 4 Department of Biostatistics & Epidemiology, Hudson College of Public Health, University of Oklahoma Health Campus, Oklahoma City, OK 73104, USA; justin-dvorak@ou.edu; 5 Department of Pediatrics, University of Arkansas for Medical Sciences, Little Rock, AR 72205, USA; tswindle@uams.edu; 6 Arkansas Children’s Research Institute, Little Rock, AR 72202, USA; 7 Arkansas Children’s Nutrition Center, Little Rock, AR 72202, USA; 8 Grand Forks Human Nutrition Research Center, Agricultural Research Services, United States Department of Agriculture, Grand Forks, ND 58203, USA; shanon.casperson@usda.gov; 9 Department of Nutritional Sciences, College of Education & Human Sciences, Oklahoma State University, Stillwater, OK 74075, USA; ashlea-braun@ouhsc.edu; 10 TSET Health Promotion Research Center, OU Health Stephenson Cancer Center, University of Oklahoma Health Campus, Tulsa, OK 74135, USA; 11 Department of Medical Imaging and Radiation Sciences, University of Oklahoma Health Sciences, Oklahoma City, OK 73117, USA; jonathan-d-baldwin@ou.edu; 12 Pediatric Dietitian Specialized in Dialysis and Renal Transplant, Oklahoma Children’s Hospital, Oklahoma City, OK 73104, USA; 13 Department of Agricultural and Human Sciences, North Carolina State University, Raleigh, NC 27695, USA; virginia_stage@ncsu.edu

**Keywords:** carotene, dietary carotenes, skin carotenoid score, reflection spectroscopy, dietary screener, Veggie Meter^®^

## Abstract

**Background**: The reflection spectroscopy (RS)-based skin carotenoid score (SCS) has been validated to assess carotenoid intake. However, associations between the SCS and individual dietary carotenoid species have been inconsistently reported in the previous literature. This study examined the utility of using a carotenoid screener along with RS to assess individual dietary carotenoid species intake among U.S. adults. **Methods**: Adults (N = 280) from eight U.S. sites completed a 44-item carotenoid intake screener to estimate total and individual dietary carotenoid intake (α-carotene, β-carotene, β-cryptoxanthin, lutein + zeaxanthin, and lycopene) over the past 7 days. Each participant’s SCS was measured using two Veggie Meter^®^ devices. Spearman’s Rank correlation tests and linear mixed models with a random intercept for site were conducted. **Results**: Mean (± Standard Deviations) carotenoid intake ranged from 21,226.8 ± 9914.1 to 28,863.5 ± 22,462.2 µg/day, and mean SCS ranged from 229.2 ± 72.3 to 325.6 ± 94.1 across the eight sites. Participants’ SCS was significantly associated with self-reported total carotenoids (mg)/week (*r_s_* = 0.2 and *B* = 0.11; *p* < 0.01) and with all individual carotenoids (*r_s_* ranged from 0.15 to 0.23, *p* < 0.01; *B* ranged from 0.11 to 21.1, *p* < 0.01), except lycopene. **Conclusions**: Our findings suggest that combining the carotenoid intake screener with RS may offer a practical approach for assessing individual carotenoid species intake in adults.

## 1. Introduction

Carotenoids are fat-soluble pigments and phytochemicals abundant in brightly colored fruits and vegetables [1,2] and have numerous health benefits [3,4,5,6,7]. The six major carotenoids in the human diet are α-carotene, β-carotene, lycopene, β-cryptoxanthin, lutein, and zeaxanthin [1]. Each carotenoid has distinct biological functions, including serving as precursors to vitamin A [1], supporting metabolic health, reducing the risk of chronic diseases, and helping prevent age-related macular degeneration and cataracts [1,5]. Despite these benefits, brightly colored vegetables, which are the primary dietary sources of carotenoids, remain among the least consumed type of vegetables in the U.S. population [8].

Data from the National Health and Nutrition Examination Survey (NHANES) 2009–2018 indicate that approximately two-thirds of total dietary carotenoids in the U.S. diet come from lycopene-rich foods, such as tomato products, salsa, pizza, and watermelon, while only 33% are derived from other carotenoid-rich sources such as carrots, spinach, kale, sweet potato, and broccoli [9]. In addition, household purchase data suggest declining trends in fruit and vegetable purchases following the COVID-19 pandemic in 2020 [10]. Together, these trends underscore the need for targeted interventions and continued monitoring of carotenoid intake in the U.S. population. However, monitoring diet is challenging because traditional dietary assessment methods are resource-intensive, time-consuming, and laborious. Consequently, objective and non-invasive approaches to assess carotenoid intake are increasingly needed.

The Veggie Meter^®^ (Longevity Link Corporation, Salt Lake City, Utah 84108, USA) is a reflection spectroscopy (RS)-based device that offers a non-invasive method to measure skin carotenoids in adults and children [11]. Skin carotenoids serve as an objective biomarker of carotenoid-rich fruit and vegetable intake over the past four to eight weeks [12,13]. The Veggie Meter^®^ has been validated against plasma carotenoids in randomized controlled trials in adults that showed a minimum increase in skin carotenoid score (SCS) of 97 units [14] following an 8-week intervention with a high-dose (31 mg/day) lycopene-rich juice and 123 units [15] following a 6-week intervention with a high-dose (8 mg/day) β-carotene-rich juice. A higher SCS has also been associated with better overall diet quality [16] and improved metabolic health indicators in adults [17,18]. These findings suggest that SCS assessment has strong potential as a non-invasive tool for dietary monitoring and health screening in community-based nutrition programs.

Although many studies reported small but positive correlations between SCSs and total dietary carotenoid intake, associations with SCSs and individual dietary carotenoids varied. For example, correlations between dietary lycopene intake and SCSs have consistently been reported as non-significant [19,20,21,22,23]. Additionally, while some studies demonstrated significant positive associations between the RS-based SCS and α-carotene, β-cryptoxanthin, lutein and zeaxanthin [19,24], others reported no association [20,21,22,23,25,26]. These inconsistencies may be due to differences in dietary assessment methods or low intake levels of certain carotenoids among study populations. Consequently, research and community nutrition programs seeking to assess individual dietary carotenoid intake may benefit from pairing SCS measurements with a targeted dietary carotenoid assessment tool that is valid for the U.S. population and is easy to administer.

Dietary screeners are brief, nutrient-focused instruments that efficiently capture targeted intake information with minimal participant burden [27]. To the authors’ knowledge, only one carotenoid screener has been developed for U.S. adults and validated within a limited population. Casperson et al. (2023) [28] developed and validated a 44-item carotenoid intake screener against corresponding plasma carotenoids (*r* = 0.35) and with the RS-based SCS (*r* = 0.24) in nonobese Midwestern American adults, where 89% were non-Hispanic White participants [28]. Faraji et al. (2022) [21] used the same carotenoid intake screener to measure dietary carotenoids in African American/Black college students and found no relationship between total dietary carotenoids and plasma carotenoids. However, Faraji et al. reported significant positive correlations (r) between SCSs and total dietary carotenoids (0.25), β-carotene (0.23), and lutein and zeaxanthin (0.28) [21]. No associations were observed for lycopene, β-cryptoxanthin, and α-carotene [21].

Taken together, existing evidence indicates that the RS-based SCS shows inconsistent associations with specific dietary carotenoids. Furthermore, the currently available brief carotenoid intake screener has been validated only within two small, region-specific populations [21,28], which limits its broader applicability. Therefore, the present study extends previous validation work by evaluating the 44-item carotenoid intake screener in a geographically diverse sample of U.S. adults. This study explored associations for individual carotenoid species to determine whether the previously reported non-significant correlations persist in a larger and more heterogeneous population. By doing so, this study provides new evidence regarding the generalizability of the screener and its utility for assessing dietary carotenoid intake in diverse U.S. adults.

## 2. Materials and Methods

### 2.1. Study Design, Recruitment, and Enrollment

This was a cross-sectional research study that was conducted across 8 different sites in the U.S. The sites were: the University of Oklahoma Health Sciences Center in Oklahoma City, OK; the University of Nebraska in Lincoln, Nebraska (UNL); East Carolina University and North Carolina State University collaborative, North Carolina (ECU/NC State); the University of Arkansas for Medical Sciences (UAMS) campuses at Fayetteville (UAMS-Fayetteville) and Little Rock (UAMS-Little Rock); Oklahoma State University in Stillwater, OK (OSU); the Grand Forks Human Nutrition Research Center in Grand Forks, North Dakota (GFHNRC); and the San Francisco Department of Public Health (SFDPH). Each site received its own IRB approval from the respective University Institutional Review Board. All sites participated in protocol development, study design, and data collection. The OUHSC was the coordinating site, and the OUHSC investigator was either added to the IRB application for all sites or completed a data transfer agreement. The original study aim was to assess inter- and intra-device reliability of a total of 16 Veggie Meter^®^ devices across these 8 sites (two devices per site), and this current research is a secondary aim of the original study [29].

### 2.2. Recruitment and Participants

Participants were recruited through word-of-mouth, online flyer distribution, and emails, until about 35 participants were recruited per site. Interested participants contacted the respective research project managers at the site, and then via email or phone conversation, an in-person visit to the laboratory setting was scheduled. Eligible participants were English-speaking, healthy adults 18 to 65 years old who had no impairments or deformities to their non-dominant ring finger. Participants were excluded or told to reschedule if they ate Doritos, Cheetos, or Takis in the previous three days prior to their appointment or used self-tanning lotion in the past two weeks prior to their data collection visit. Recruitment and data collection occurred from July 2022 through November 2023, and participants in some sites received financial compensation of varying amounts ($15, $25, and $35) based on the site-level funding availability and IRB approvals.

### 2.3. Procedure

Participants visited the research data collection sites one time. During the data collection visits to the laboratory settings, research team members collected signed consents from participants. After collecting consents, participants’ height and weight data were collected at 5 sites. At two research sites, SFDPH and UAMS-Little Rock, participants’ self-reported height and weight were collected; the UAMS-Fayetteville site did not collect any anthropometric data. Then, participants completed online surveys using Qualtrics on a laptop or iPad at 5 sites or in paper–pencil modality at three sites: OSU, GFNHRC, and SFDPH. The total time commitment for completing these three questionnaires was 30 to 50 min for the participants. During this single visit to the research lab, the research team members collected participants’ SCSs using two Veggie Meter^®^ devices. Below is a more detailed protocol for each of these measures.

### 2.4. Anthropometric Measures

At the 5 research sites that measured participants’ height and weight, height was measured in bare or socked feet to the nearest half centimeter (cm) and weight was measured to the nearest 0.01 kg. Body Mass Index (BMI) was calculated from corrected height and weight using the formula BMI = (Weight in kg)/(Height in m)^2^. Further details about height–weight measurement are reported elsewhere [29].

### 2.5. Online Questionnaire and Survey

Participants filled out three online questionnaires in total. The first questionnaire asked about participants’ demographic characteristics, such as age, sex, race/ethnicity, and smoking status. The second questionnaire asked about participants’ dietary intake of carotenoid-rich foods over the last week using a short 44-item dietary carotenoid intake screener developed and validated by Casperson et al. (2023) [28] and Faraji et al. (2022) [21]. In the current study, it took about 20 min for participants to complete this screener. The USDA-ARS GFHNRC provided values of micrograms of total dietary carotenoids, β-carotene, α-carotene, β-cryptoxanthin, lycopene, lutein and zeaxanthin provided per serving of each food in the screener [21,28]. Total 7-day intake for each carotenoid was calculated by multiplying the carotenoid content (µg/serving) of each food by the number of servings consumed and summing across all foods.

The third questionnaire asked participants about their skin reactivity, using the Fitzpatrick scale [30], which is a reliable and valid self-report tool [31]. This tool is considered the gold standard for assessing skin reactivity to the sun’s ultraviolet radiation and the skin’s ability to tan and is highly correlated with skin melanin content (*r_s_* = 0.98, *p* < 0.001) [32]. In the current study, this data was collected as an alternative indicator of skin pigmentation or melanin content.

### 2.6. Skin Carotenoid Score (SCS)

The original study aimed to capture inter-device reliability of the Veggie Meter^®^ devices. Detailed protocols for the SCS data collection for each site have been reported [29]. We collected a total of 10 scans using two Veggie Meter^®^ devices (Device 1 and Device 2) for each participant per site (8 sites; 16 devices total). Device numbering was arbitrary and determined independently by data collectors at each site. During data collection, the Veggie Meter^®^ devices were calibrated based on the manual provided by the company (Longevity Link Corporation, Salt lake City, Utah 84108, USA) [33]. After calibrating, participants were asked to wash and dry their hands, then place their non-dominant ring finger on the lens to collect the SCS. Participants inserted and removed their fingers for each scan, with a five-minute gap between each scan until 5 non-consecutive SCSs were collected from each device. Every site used a unique pair of Veggie Meter^®^ devices to collect the data. For each pair of scans, the order of the devices was counterbalanced. Researchers immediately recorded these SCSs on a paper-based form and later filled out an online Qualtrics form to merge all datasets from the 8 sites. Between the two devices at each site intra-class correlation coefficients ranged from 0.58 to 0.94, and absolute relative differences ranged from 12.7% to 22% [29]. Due to variability in inter-device reliability across sites, we analyzed SCS values separately by device position. Data from Device 1 across sites were pooled to compute correlations, and the same was done for every Device 2, yielding two sets of correlations based on eight site-specific device pairs.

### 2.7. Data Analysis

Datasets were checked for erroneous values, missingness, and duplicates. Values of “Prefer not to answer” or “Don’t know/not sure” were treated as missing. About 0.4–1.8% of data were missing across the variables, except marital status (12.86%) and BMI data for the UAMS-Fayetteville. Participants’ average SCS was calculated for each set of devices.

Given that prior studies reported bivariate correlations between the SCS and dietary carotenoids, Spearman’s Rank correlation analyses were done. First, random separate correlation analyses were conducted for each set of devices. Additionally, linear mixed-effects regression models were conducted to examine associations between individual predictors and participant-level SCS, controlling for BMI for each set of devices. To test the significance of each predictor, the full model was compared with a reduced model that excluded the predictor of interest, using Type III likelihood ratio tests to calculate *p*-values. Each regression model included a random intercept to account for clustering by site. The units for dietary carotenoids were set as milligrams (mg) in the correlation and regression models. Complete-case analysis was used for all statistical models.

Adjusted *p*-values were calculated for multiple correlation analyses and comparisons using the Bonferroni method for all predictors, where the number of predictors k = 60 across all models run. All data analyses were conducted using R v4.5.0.

## 3. Results

Demographic characteristics and descriptive statistics are provided in Table 1. The total number of participants included in the current analyses was 280, and the number of participants per site ranged from 33 to 37. The majority were female (72.4%). About 9.8% of the participants self-reported their race as African American, 1.5% American Indian or Alaska Native, 19.3% Asian, 0.4% Pacific Islander, 68% White, and 1.1% from other racial identities. About 8.6% of the participants reported Hispanic ethnicity. The majority of participants (81.7%) were college graduates or higher. Participants’ ages ranged from 18 to 70 years, with a mean age of 31.7 (±10.2) years. About 94% reported that they did not currently smoke and/or use any vaping or electronic smoking devices. Participants fell into several Fitzpatrick scale score categories, with most (74.7%) being either Type III, Medium White to Olive, or Type IV, Olive Tone (Table 1). Participants’ mean BMI was 26.96 ± 6.03 kg/m^2^, and 4.4% of the participants were underweight; 40.6% had normal weight; 27.7% had overweight; and 27.3% had obesity.

For 90% of participants, we measured the SCS by scanning their left ring finger. Participants’ mean SCS for device set 1 ranged from 229.2 ± 72.3 to 325.6 ± 94.1, and for device set 2, the mean SCS ranged from 241 ± 65.6 to 304.9 ± 87.1. The mean (±SD) SCS from each device per site is provided in Table 1. The mean (±SD) dietary carotenoid intake across the sites for all participants is reported in Table 2. The mean total dietary carotenoid intake was 24,739.2 ± 16,073 (mcg/week). Participants’ mean dietary intake for each food on the carotenoid intake screener is provided in Supplementary File S1.

Spearman’s Rank correlations between the SCS and total dietary carotenoids for device set 1 was *r_s_*_1_ = 0.21, *p* < 0.001 and for device set 2 was *r*_*s*2_ = 0.17, *p* = 0.004. The SCS was also correlated with dietary α-carotene (*r*_*s*1_ = 0.22, *p* < 0.001; *r*_*s*2_ = 0.21, *p* < 0.001), β-carotene (*r*_*s*1_ = 0.25, *p* < 0.001; *r*_*s*2_ = 0.21, *p* < 0.001), β-cryptoxanthin (*r*_*s*1_ = 0.21, *p* < 0.001; *r*_*s*2_ = 0.23, *p* < 0.001), and lutein and zeaxanthin (*r*_*s*1_ = 0.21, *p* < 0.001; *r*_*s*2_ = 0.15, *p* < 0.001). Correlation between lycopene and SCSs was not significant (*r*_*s*1_ = −0.01, *p* = 0.8; *r*_*s*2_ = 0.005, *p* =0.9).

Regression results were similar to Spearman’s Rank correlations. For both sets of devices, among the demographic characteristics, Fitzpatrick skin type, smoking habits, and other non-dietary variables, only BMI was significantly correlated with SCSs. A higher BMI was associated with a lower SCS (*B*_1_ = −4.2, *p* < 0.0001; *B*_2_ = −4, *p* < 0.0001). After adjusting for multiple comparisons and controlling for BMI, α-carotene (*B*_1_ = 2.11, *p* < 0.001; *B*_2_ = 2.19, *p* < 0.001), β-carotene (*B*_1_ = 0.47, *p* < 0.001; *B*_2_ = 0.43, *p* < 0.001), β-cryptoxanthin (*B*_1_ = 20.5, *p* = 0.004; *B*_2_ = 21.1, *p* = 0.004), and total carotenoids (*B*_1_ = 0.13, *p* = 0.014; *B*_2_ = 0.11, *p* = 0.045) remained significantly associated with SCSs. After adjusting for multiple analyses, lutein and zeaxanthin were significantly correlated with SCSs from device set 1 but not with device set 2 (*B*_1_ = 0.3, *p* = 0.008; *B*_2_ = 0.24, *p* = 0.01). Lycopene was not associated with SCSs in either device set (*B*_1_ = −0.13, *p* = 1, *B*_2_ = −0.11, *p* = 1.0). All bivariate correlation statistics, regression model coefficients, and corresponding *p*-values are reported in Table 3. Additionally, marginal and conditional R^2^ values and effect size measures for mixed models are provided in Supplementary File S2.

## 4. Discussion

The current multisite study explored the relationship between the RS-based SCS and self-reported dietary carotenoid intake measured using a dietary screener [21,28] and determined the utility of combining the carotenoid intake screener with RS. This was a secondary aim analysis of a previously published study, where researchers used two sets of Veggie Meter^®^ devices to collect participants’ SCSs. The current study showed slight variation in how participants’ SCSs were associated with total dietary carotenoids and individual carotenoids. The observed correlations were significant but modest (*r_s_* ≈ 0.15–0.25), corresponding to approximately 3–6.4% shared variances. Additionally, BMI was significantly related to SCSs, but Fitzpatrick skin type, smoking status and other demographic factors were not related to RS-based SCSs in this sample. Collectively, these findings add to the evidence supporting the criterion-related validity and generalizability of the 44-item carotenoid intake screener across a broader U.S. adult population than previously studied. In addition, they provide new evidence that inter-device variability among Veggie Meter^®^ devices may affect the strength of associations between SCSs and individual dietary carotenoids, underscoring the importance of standardized device calibration and the complementary use of dietary assessment tools when estimating individual carotenoid intake in research settings.

As this was a secondary analysis, the available dataset limited the ability to consider many potentially relevant covariates that may have influenced the outcomes. Among the variables considered, smoking habits were not associated with SCSs in the current study. This may be because approximately 95% of participants in the current study were non-smokers, compared to about 65% in a previous study that reported a significant association [34]. Similarly, the majority of the participants were highly educated, with 95.3% having some college or graduate degrees. This potentially limited the statistical power to detect effects of smoking and education in the current sample. Consistent with prior research, there was a negative association between SCSs and BMI, likely due to the sequestering of the circulating carotenoids in adipose tissue among individuals with higher body fat [5,35,36]. The original validation study of the carotenoid intake screener did not include participants with obesity; however, in the current study, 27% of participants had obesity. Thus, the current study extends the screener’s usability among individuals with higher BMI.

Regarding correlations between individual dietary carotenoids and SCSs, the results were also broadly consistent with prior studies. Prior studies have demonstrated small-to-moderate correlations between total self-reported dietary carotenoid and SCSs (*r* = 0.17–0.69) reported in several studies [19,25,28,37,38,39,40,41], which is comparable to the current study findings (*r_s_* = 0.17 to 0.21 and *B* = 0.11 to 0.13). Evidence for associations between individual dietary carotenoids and the RS-based SCSs has been more limited. In previous research, results for lycopene, β-cryptoxanthin, lutein and zeaxanthin varied across studies [19,25,42,43]. In the current study, all self-reported intake of individual carotenoids except lycopene was significantly correlated with SCSs in Device 1.

Despite relatively high lycopene intake (approximately 36% of total carotenoid intake), lycopene was not correlated with SCSs. Similarly, a 3-week intervention with lycopene-rich juice in adults with obesity increased plasma lycopene but did not change the SCS [44]. Wu et al. also reported no significant correlation between plasma lycopene and the Veggie Meter^®^ SCS, suggesting that the RS-based SCS may not reliably detect lycopene [19]. In contrast, Kiem et al. found a small but positive correlation (*r_s_* = 0.2, *p* < 0.001) between plasma lycopene and the Veggie Meter^®^ SCS. These inconsistent findings suggest that further research is needed to explore the reliability of the Veggie Meter^®^ in detecting lycopene in skin [43]. The RS-based SCS should be interpreted cautiously in populations with lycopene-rich diets (e.g., high consumption of tomatoes or watermelon).

There was high inter-device variability between the two sets of devices (absolute relative difference 7.5–22%) [29]. Device variability may have attenuated associations between the SCS and specific dietary carotenoids, particularly for carotenoids that exhibit weaker relationships with skin carotenoid concentrations. These findings highlight the importance of considering inter-device variability when interpreting RS-based SCS measurements across studies or devices. As evidence of variability among Veggie Meter^®^ devices continues to accumulate [45,46], combining the RS-based SCS with a dietary assessment tool, such as a carotenoid intake screener, may provide a more robust assessment of individual carotenoid intake than relying on either measure alone.

A methodological contribution of the current study is demonstrating that the carotenoid intake screener [28] can capture carotenoid intake patterns in U.S. populations over the past week with relatively low participant burden, requiring approximately 20 min to complete. Additionally, researchers can estimate total and individual dietary carotenoid intake by multiplying the reported servings of foods and beverages by their corresponding carotenoid values per serving. By focusing specifically on carotenoid-rich foods, the screener may provide a more direct and reliable estimate of carotenoid intake compared to methods designed to capture overall dietary patterns, while reducing measurement error. Finally, consistent with prior work, current study findings suggest that Fitzpatrick skin type (I to V) did not modify associations between dietary carotenoids and the SCS [19,21]. This supports the broader applicability of the RS-based SCS across individuals with diverse skin types.

### Strengths and Limitations

This study has several strengths. The multisite design and use of eight different sets of Veggie Meter^®^ devices enhance the generalizability of findings. Researchers in the current study considered a comprehensive list of demographic variables, smoking habits, varied obesity status, and skin types further, which allowed for the exploration of potential covariates. Additionally, large variability in the data and standard deviations are expected with a single data collection timepoint for dietary assessment [47]. However, the carotenoid intake screener’s 7-day reference period may have better captured habitual intake of carotenoid-rich foods than two 24 h recalls [47], which are widely used for monitoring dietary intake in the U.S.

The current study is a secondary analysis of the original study [29]; therefore, data on several potential covariates of SCSs could not be included in the analyses. For example, dietary fat intake, physical activity, and seasonality may be associated with SCSs, but these data were not collected in the current study, although some confounders were statistically adjusted in the models. However, because of the cross-sectional nature of the dataset, residual confounding cannot be excluded entirely. Another limitation is that carotenoid-containing dietary supplement use was not assessed. Supplemental carotenoid intake may increase skin carotenoid scores independently of dietary intake and therefore represents a potential source of residual confounding. Consequently, some participants may have had an elevated SCS that was not attributable solely to dietary carotenoid intake. In addition, self-reported dietary intake remains subject to recall error and misreporting. While the screener captures both total and specific carotenoids, it may not fully reflect differences in food sources or carotenoid bioavailability. Among other limitations, the current sample was a convenience sample and included predominantly female and highly educated participants. Although ethnic diversity is included in the sample, the current sample was not designed to be nationally representative. As a result of the sampling strategy in the original study, Hispanic adults are under-represented and the Fitzpatrick skin types in the current sample under-represent individuals at the Type I and VI categories [48]. The findings should be interpreted with consideration of these limitations.

## 5. Conclusions

Consistent with prior research, the current study findings align with the growing body of evidence that SCSs may be a reliable biomarker of carotenoid exposure overall but not of lycopene. The current study findings provide additional evidence for the criterion-related validity of the carotenoid intake screener and highlight the value of combining dietary assessment tools with RS-based SCS measures to improve the methodological rigor of future research studies in U.S. adults.

## Figures and Tables

**Table 1:** Demographic characteristics and descriptive statistics for a geographically diverse U.S. sample recruited from eight different research sites (N = 280).

Characteristics/Variables	N (%) ^1^	
Sex		
Male	77 (27.6%)	
Female	202 (72.4%)	
Race		
African American or Black	27 (9.8%)	
American Indian or Alaska Native	4 (1.5%)	
Asian	53 (19.3%)	
Pacific Islander	1 (0.4%)	
White	187 (68%)	
Other	3 (1.1%)	
Ethnicity		
Hispanic or Latino	24 (8.6%)	
Non-Hispanic	255 (91.4%)	
Education		
High school/GED	13 (4.7%)	
Some college or tech school (1–3 yrs)	38 (13.6%)	
College graduate	115 (41.2%)	
Professional or graduate school	113 (40.5%)	
Marital Status		
Single	106 (43.4%)	
Living with partner (unmarried)	27 (11.1%)	
Married	101 (41.4%)	
Separated	3 (1.2%)	
Divorced	7 (2.9%)	
Smoked at least 100 cigarettes in your entire life?		
Yes	43 (15.6%)	
No	232 (84.4%)	
How often do you currently smoke?		
Every day	2 (0.7%)	
Some days	13 (4.7%)	
Not at all	260 (94.6%)	
How often do you currently use chewing tobacco, snuff, or snus?		
Every day	0 (0)	
Some days	1 (0.4%)	
Not at all	276 (99.6%)	
How often do you currently use e-cigarettes, a vape pen, or an e-hookah?		
Every day	6 (2.2%)	
Some days	11 (4%)	
Not at all	260 (93.9%)	
Finger used for skin carotenoid score		
Left ring finger	252 (90%)	
Right ring finger	28 (10%)	
Fitzpatrick scale score categories		
Type I (0–6); Light, Pale White	7 (2.5%)	
Type II (7–13); White, Fair	45 (16.3%)	
Type III (14–20); Medium White to Olive	112 (40.4%)	
Type IV (21–27); Olive Tone	95 (34.3%)	
Type V (28–34); Light Brown	18 (6.5%)	
Type VI (35–40); Dark Brown	0 (0)	
	Mean ± SD	
Age	31.69 ± 10.17	
Height (in cm)	167.72 ± 9.48	
Weight (in kg)	75.87 ± 18.14	
Body Mass Index (BMI) (=kg/m^2^)	26.96 ± 6.03	
Mean skin carotenoid score (SCS)	282.97 ± 26.47	
Mean SCS per site ^2^	Device 1	Device 2
OUHSC	291.34 ± 90.04	296.57 ± 86.29
UNL	289.73 ± 74.29	280.77 ± 72.35
ECU/NC state	274.47 ± 60.60	241.03 ± 65.55
GNHNRC	276.61 ± 83.86	277.05 ± 85.28
UAMS-Fayetteville	293.64 ± 60.11	300.69 ± 62.92
UAMS-Little Rock	229.17 ± 72.34	243.50 ± 74.09
SFDPH	325.63 ± 94.08	295.01 ± 99.93
OSU	307.30 ± 87.84	304.94 ± 87.13

^1^ Percentages were calculated using available (non-missing) data; therefore, denominators may differ across variables and Percentages may not total 100% because of rounding to the nearest decimal place. ^2^ Participants’ skin carotenoid score (SCS) was collected using two Veggie Meter^®^ devices (Device 1 and Device 2). There were a total of 8 sites and 16 Veggie Meter^®^ devices. For each participant, a total of 10 scans were collected.

**Table 2:** Mean dietary carotenoid intake (mcg) for 7 days across 8 research sites measured using the carotenoid intake screener in a geographically diverse U.S. sample (N = 278) ^1^.

Carotenoids	Total	OUHSC	UNL	ECU/ NC State	GNHNRC	UAMS-Fayetteville	UAMS-Little Rock	SFDPH	OSU
α-Carotene	933.8 ± 1242.1	703.9 ± 702.4	1009.6 ± 878.7	580.8 ± 452.6	1123.4 ± 1378	670 ± 556.3	803.5 ± 711.4	1844.2 ± 2617.6	720.06 ± 582.90
β-Carotene	7439.7 ± 6336.7	7239.1 ± 7066	7791.7 ± 7000.7	6823 ± 4975.5	6872.3 ± 5257.9	5943.7 ± 3909.7	7513.2 ± 6424.6	10,377.7 ± 7746.2	6871.3 ± 6916.7
β-Cryptoxanthin	137.3 ± 111.6	114.4 ± 96.9	163.4 ± 144.9	96.2 ± 104.7	134.2 ± 105.2	152 ± 97.4	156.1 ± 124.4	162.5 ± 110.4	120 ± 88.7
Lutein + zeaxanthin	7416.5 ± 7311.9	8668.3 ± 10,263	7893.2 ± 9110.9	7411.2 ± 7456.3	5439.4 ± 4457	6381.7 ± 5684.4	6679 ± 5905	10,301.5 ± 7166.5	6498.4 ± 6183.6
Lycopene	8811.9 ± 6453.1	8026.1 ± 3720.9	12,005.3 ± 9345.9	7787.6 ± 5386.6	9100.4 ± 7205.3	8079.4 ± 4773	8907.1 ± 5286.9	9088.3 ± 6208.8	7459.4 ± 7375.9
Total carotenoids	24,739.2 ± 16,073	24,751.9 ± 17,833.5	28,863.5 ± 22,462.2	22,698.7 ± 13,514.2	22,670 ± 12,091.6	21,226.8 ± 9914.1	24,059.1 ± 12,353	31,774.3 ± 17,903.1	21,669 ± 16,981

^1^ Carotenoid intake screener is a validated 44-item dietary screener for assessing total and individual dietary carotene in U.S. populations [28,29].

**Table 3:** Associations between dietary carotenoid intake and skin carotenoid score measured using two sets of Veggie Meter^®^ devices in a geographically diverse U.S. sample.

Dietary Carotenoid Intake over 1 Week (mg)	Spearman’s Rank Correlation ^1^ (*n* = 278)		Linear Mixed Models (*n* = 245)			
			Regression Coefficients ^1,3,4^ Unstandardized, *B* (Standardized, *β*)	95% Confidence Interval ^5^		*p*-Values ^6^
	*r_s_*	*p*-Values ^2^		Lower Limit	Upper Limit	
Set of Device 1						
α-Carotene	0.22	**0.0003**	2.11 (0.23)	1.06	3.16	**0.0001**
β-Carotene	0.25	**0.0000**	0.47 (0.26)	0.27	0.67	**<0.0001**
β-Cryptoxanthin	0.21	**0.0004**	20.5 (0.19)	8.6	32.39	**0.0007**
Lutein and zeaxanthin	0.21	**0.0004**	0.3 (0.18)	0.11	0.48	**0.0013**
Lycopene	−0.01	0.8251	−0.13 (−0.07)	−0.34	0.08	0.2198
Total carotenoids	0.21	**0.0006**	0.13 (0.18)	0.05	0.21	**0.0022**
Set of Device 2						
α-Carotene	0.21	**0.0004**	2.19 (0.24)	1.13	3.25	**0.0001**
β-Carotene	0.21	**0.0005**	0.43 (0.24)	0.23	0.64	**<0.0001**
β-Cryptoxanthin	0.23	**0.0002**	21.14 (0.20)	9.05	33.22	**0.0006**
Lutein and zeaxanthin	0.15	**0.0099**	0.24 (0.15)	0.05	0.42	0.0125
Lycopene	0.005	0.9385	−0.11 (−0.06)	−0.32	0.11	0.3250
Total carotenoids	0.17	**0.0039**	0.11 (0.16)	0.03	0.2	**0.0076**

^1^ Dependent variable = average skin carotenoid score (SCS). ^2^ Bolded *p*-values from Spearman’s rank correlations remained significant after adjusting for multiple comparisons. Adjusted *p*-values were computed using the Bonferroni method where total number of predictors were k = 6). ^3^ Model covariates included Body Mass Index (BMI, kg/m^2^). ^4^ Total carotenoid and individual carotenoid species intakes over 7 days (mg) were used for this analysis. Unstandardized Regression Coefficients (B) should be interpreted as the unit increase in dietary carotenoid intake associated with 1 unit change in the skin carotenoid score (measured by the Veggie Meter^®^). For example, a 4.7 mg/week increase in β-carotene intake corresponds to a 10-unit increase in the SCS, after adjusting for BMI. Again, a 210 mg/week increase in β-cryptoxanthin intake corresponds to a 10-unit increase in the SCS, after adjusting for BMI. ^5^ CI values are for Unstandardized Regression Coefficients. ^6^ Bolded *p*-values are significant after adjusting for multiple analyses using the Bonferroni method, where the total number of predictors k = 60 across all models.

## Data Availability

Deidentified dataset can be available upon further IRB approval, if requested.

## Supplementary Materials

The following supporting information can be downloaded at https://www.mdpi.com/article/10.3390/nu18152515/s1. Supplementary File S1: Participant’s individual food and beverage consumption over the past 7 days reported using the carotenoid intake screener (*n* = 278). Supplementary File S2: Standardized Regression Coefficients, marginal and conditional R^2^ values, and effect size measures for mixed models represented in Table 3.

## Abbreviations

The following abbreviations are used in this manuscript:

SCS	Skin Carotenoid Score
RS	Reflection Spectroscopy
BMI	Body Mass Index
NHANES	National Health and Nutrition Examination Survey
OUHSC	University of Oklahoma Health Sciences Center
UNL	University of Nebraska in Lincoln
ECU	East Carolina University
NC	North Carolina
UAMS	University of Arkansas for Medical Sciences
OSU	Oklahoma State University in Stillwater
GFHNRC	Grand Forks Human Nutrition Research Center in Grand Forks
SFDPH	San Francisco Department of Public Health

## References

[1] Office of Dietary Supplements Vitamin A and Carotenoids. https://ods.od.nih.gov/factsheets/VitaminA-HealthProfessional/.

[2] Carotenoids. https://lpi.oregonstate.edu/mic/dietary-factors/phytochemicals/carotenoids.

[3] Aune D., Chan D.S., Vieira A.R., Navarro Rosenblatt D.A., Vieira R., Greenwood D.C., Norat T. (2012). Dietary Compared with Blood Concentrations of Carotenoids and Breast Cancer Risk: A Systematic Review and Meta-Analysis of Prospective Studies. Am. J. Clin. Nutr..

[4] Beydoun M.A., Chen X., Jha K., Beydoun H.A., Zonderman A.B., Canas J.A. (2019). Carotenoids, Vitamin A, and Their Association with the Metabolic Syndrome: A Systematic Review and Meta-Analysis. Nutr. Rev..

[5] Moran N.E., Mohn E.S., Hason N., Erdman J.W., Johnson E.J. (2018). Intrinsic and Extrinsic Factors Impacting Absorption, Metabolism, and Health Effects of Dietary Carotenoids. Adv. Nutr..

[6] Sumalla-Cano S., Eguren-García I., Lasarte-García Á., Prola T.A., Martínez-Díaz R., Elío I. (2024). Carotenoids Intake and Cardiovascular Prevention: A Systematic Review. Nutrients.

[7] Yu X., Yuan C., Song X., Zhang H. (2023). Association of Dietary Carotenoids Intakes with Obesity in Adults: NHANES 2007–2018. J. Nutr. Sci. Vitaminol..

[8] Ansai N., Wambogo E.A. (2021). Fruit and Vegetable Consumption Among Adults in the United States, 2015–2018.

[9] Kim K., Madore M.P., Chun O.K. (2024). Changes in Intake and Major Food Sources of Carotenoids among U.S. Adults between 2009–2018. Metabolites.

[10] Okrent A., Zeballos E.U.S. (2025). Household Food Spending Post COVID-19 and the Implications for Diet Quality.

[11] Longevity Link Corporation. https://longevitylinkcorporation.com.

[12] Ermakov I.V., Ermakova M., Sharifzadeh M., Gorusupudi A., Farnsworth K., Bernstein P.S., Stookey J., Evans J., Arana T., Tao-Lew L. (2018). Optical Assessment of Skin Carotenoid Status as a Biomarker of Vegetable and Fruit Intake. Arch. Biochem. Biophys..

[13] Jahns L., Johnson L.K., Mayne S.T., Cartmel B., Picklo M.J., Ermakov I.V., Gellermann W., Whigham L.D., Picklo M.J., Johnson L.K. (2014). Skin and Plasma Carotenoid Response to a Provided Intervention Diet High in Vegetables and Fruit: Uptake and Depletion Kinetics. Am. J. Clin. Nutr..

[14] Casperson S.L., Roemmich J.N., Larson K.J., Hess J.M., Palmer D.G., Jahns L. (2023). Sensitivity of Pressure-Mediated Reflection Spectroscopy to Detect Changes in Skin Carotenoids in Adults Without Obesity in Response to Increased Carotenoid Intake: A Randomized Controlled Trial. J. Nutr..

[15] Jilcott Pitts S.B., Moran N.E., Laska M.N., Wu Q., Harnack L., Moe S., Carr-Manthe P., Gates E., Chang J., Zaidi Y. (2023). Reflection Spectroscopy-Assessed Skin Carotenoids Are Sensitive to Change in Carotenoid Intake in a 6-Week Randomized Controlled Feeding Trial in a Racially/Ethnically Diverse Sample. J. Nutr..

[16] Sisson S.B., Kasahara E., Casperson S., Pitts S.J., Reese J., Zhang Y., Taniguchi T., Clyma K.R., Hayman J., Jernigan V.B.B. (2025). Association of Veggie Meter^®^-Assessed Skin Carotenoids and Dietary Intake Among Indigenous Families: The ISA “Go Healthy” Study. Curr. Dev. Nutr..

[17] Kimura Y., Hata J., Shibata M., Honda T., Sakata S., Furuta Y., Oishi E., Kitazono T., Ninomiya T. (2024). Skin Carotenoid Scores and Metabolic Syndrome in a General Japanese Population: The Hisayama Study. Int. J. Obes..

[18] Obana A., Nakamura M., Miura A., Nozue M., Muto S., Asaoka R. (2024). Association between Atherosclerotic Cardiovascular Disease Score and Skin Carotenoid Levels Estimated via Refraction Spectroscopy in the Japanese Population: A Cross-Sectional Study. Sci. Rep..

[19] Wu Q., Cherry C.W., Jilcott Pitts S., Laska M.N., Craft N., Moran N.E. (2025). A Reflection-Spectroscopy Measured Skin Carotenoid Score Strongly Correlates with Plasma Concentrations of All Major Dietary Carotenoid Species except for Lycopene. Nutr. Res..

[20] Varghese V., Cepni A.B., Chang J., Kim H., Moran N.E., Ledoux T.A. (2024). Skin Carotenoids Measured by Reflection Spectroscopy Correlate With Dietary Carotenoid Intake in Racially and Ethnically Diverse US Toddlers From Houston, Texas. J. Acad. Nutr. Diet..

[21] Faraji B., Bukowski M.R., Thompson-Johnson T., Krusinski L., Goldberg J.L., Brooks C.M., Snyder S. (2022). Skin Carotenoid Status of Black/African American College Students Correlates with Plasma Carotenoids and Fruit and Vegetable Intake Independent of Skin Tone. Int. J. Clin. Nutr. Diet..

[22] Rosok L.M., Fifield L.M., Sarma R., Keye S.A., Walk A.M., Khan N.A. (2025). Relations between Dietary and Skin Carotenoids across 12 Months in a Midwestern Sample of Toddlers. Nutr. Res..

[23] Liu R., Rosok L.M., Black M., Erdman J.W., Khan N.A. (2026). Reflection Spectroscopy Skin Carotenoids Correlate with Serum Carotenoids in School-Aged Children. J. Nutr..

[24] Henning T., Wagner P., Gedat E., Kochlik B., Kusch P., Sowoidnich K., Vastag M., Gleim J., Braune M., Maiwald M. (2023). Evaluation of Modern Approaches for the Assessment of Dietary Carotenoids as Markers for Fruit and Vegetable Consumption. Nutrients.

[25] Sklar E., Radtke M.D., Steinberg F.M., Medici V., Fetter D.S., Scherr R.E. (2025). Nutrition Knowledge, Food Insecurity, and Dietary Biomarkers: Examining Fruit and Vegetable Intake Among College Students. Nutrients.

[26] Keller J.E., Taylor M.K., Smith A.N., Littrell J., Spaeth K., Boeckman C.R., Burns J.M., Sullivan D.K. (2022). Correlation of Skin Carotenoid Content with 3-Day Dietary Intake in Community Dwelling Older Adults. J. Food Compos. Anal..

[27] Dietary Screener Questionnaire in the NHANES 2009-10: Background. EGRP/DCCPS/NCI/NIH. https://epi.grants.cancer.gov/nhanes/dietscreen/.

[28] Casperson S.L., Scheett A., Palmer D.G., Jahns L., Hess J.M., Roemmich J.N. (2023). Biochemical Validation of a Self-Administered Carotenoid Intake Screener to Assess Carotenoid Intake in Nonobese Adults. Curr. Dev. Nutr..

[29] Sisson S.B., Helms E., Casperson S., Hasnin S., Pitts S.J., Stage V.C., Long C.R., Massey-Swindle T., Dev D.A., Braun A. (2025). Examining Intra- and Inter-Device Reliability of Pressure-Mediated Reflection Spectroscopy in a Multi-State Sample of Healthy Adults. Public Health Nutr..

[30] Fitzpatrick T.B. (1988). The Validity and Practicality of Sun-Reactive Skin Types I Through VI. Arch. Dermatol..

[31] Roberts W.E. (2009). Skin Type Classification Systems Old and New. Dermatol. Clin..

[32] Wilkes M., Wright C.Y., du Plessis J.L., Reeder A. (2015). Fitzpatrick Skin Type, Individual Typology Angle, and Melanin Index in an African Population: Steps Toward Universally Applicable Skin Photosensitivity Assessments. JAMA Dermatol..

[33] Longevity Link. https://longevitylinkcorporation.com/products.

[34] Augimeri G., Lofaro D., Vivacqua A., Barone I., Giordano C., Morelli C., Conforti D., Sisci D., Catalano S., Bonofiglio D. (2025). Associations among Skin Carotenoids, Anthropometric Parameters and Healthy Lifestyle Behaviors in Young Adults: A Cross-Sectional, Population-Based Study. J. Transl. Med..

[35] Madore M.P., Hwang J.-E., Park J.-Y., Ahn S., Joung H., Chun O.K. (2023). A Narrative Review of Factors Associated with Skin Carotenoid Levels. Nutrients.

[36] Radtke M.D., Pitts S.J., Jahns L., Firnhaber G.C., Loofbourrow B.M., Zeng A., Scherr R.E. (2020). Criterion-Related Validity of Spectroscopy-Based Skin Carotenoid Measurements as a Proxy for Fruit and Vegetable Intake: A Systematic Review. Adv. Nutr..

[37] Moran N.E., Chang J., Stroh R., Zaidi Y., Hason N., Musaad S., O’Connor T. (2022). Noninvasive Reflection Spectroscopy Measurement of Skin Carotenoid Score in Infants Is Feasible and Reliable. J. Nutr..

[38] Pickett-Nairne K., Glueck D., Thomson J., Weiss R., Fuller K.N.Z., Fabbri S., Schaefer C., Evans C., Bowhay E., Martinez M. (2024). Validation of a Food Frequency Questionnaire: VioScreen-Allergy. Nutrients.

[39] Radtke M.D., Chodur G.M., Bissell M.C.S., Kemp L.C., Medici V., Steinberg F.M., Scherr R.E. (2023). Validation of Diet ID^TM^ in Predicting Nutrient Intake Compared to Dietary Recalls, Skin Carotenoid Scores, and Plasma Carotenoids in University Students. Nutrients.

[40] Amoah I., Cairncross C., Rush E. (2023). Vegetable-Enriched Bread: Pilot and Feasibility Study of Measurement of Changes in Skin Carotenoid Concentrations by Reflection Spectroscopy as a Biomarker of Vegetable Intake. Food Sci. Nutr..

[41] Jahns L., Johnson L.K., Conrad Z., Bukowski M., Raatz S.K., Jilcott Pitts S., Wang Y., Ermakov I.V., Gellermann W. (2019). Concurrent Validity of Skin Carotenoid Status as a Concentration Biomarker of Vegetable and Fruit Intake Compared to Multiple 24-h Recalls and Plasma Carotenoid Concentrations across One Year: A Cohort Study. Nutr. J..

[42] Fam V.W., Holt R.R., Hackman R.M., Keen C.L., Sivamani R.K. (2020). Prospective Evaluation of Mango Fruit Intake on Facial Wrinkles and Erythema in Postmenopausal Women: A Randomized Clinical Pilot Study. Nutrients.

[43] Kiem F., Lackner S., Meier-Allard N., Schimpel C., Mörkl S., Unterweger T., Gruber J., Strobl H., Holasek S. (2026). Validation of Noninvasive Cutaneous Carotenoid Measurements as Nutritional Biomarker Across Body Composition Groups. Food Sci. Nutr..

[44] Jilcott Pitts S.B., Wu Q., Laska M.N., Gates E., Seese M.H., Senkus K.E., Carrero Longlax S., Portillo-Varela A., DiNardo A.R., Moran N.E. (2025). Daily Intake of a Lycopene-Rich Juice Is Associated with Reductions in Inflammatory Markers but Not Increases in Skin Carotenoids in a Pilot Study among Participants with Obesity. BMC Nutr..

[45] Hasnin S., Pitts S.B.J., Roman C.K., Khan N.A. (2026). Skin Carotenoid Score Level and Race Affect Intradevice Repeatability of Veggie Meter^®^ at a Single Time Point. J. Nutr. Educ. Behav..

[46] Obana A., Asaoka R., Takayanagi Y., Gohto Y. (2023). Inter-Device Concordance of Veggie Meter—A Reflection Spectroscopy to Measure Skin Carotenoids. J. Biophotonics.

[47] Willett W. (2012). Nutritional Epidemiology.

[48] He S.Y., McCulloch C.E., Boscardin W.J., Chren M.-M., Linos E., Arron S.T. (2014). Self-Reported Pigmentary Phenotypes and Race Are Significant but Incomplete Predictors of Fitzpatrick Skin Phototype in an Ethnically Diverse Population. J. Am. Acad. Dermatol..

