# Supplementary material for: Self-Reported Dietary Carotenoid Intake and Skin Carotenoid Score Status in a Geographically Diverse U.S. Sample
Source: Nutrients. 2026 Aug 3;18(15):2515. doi: 10.3390/nu18152515 (PMC13467704; doi:10.3390/nu18152515)
Supplement: Supplementary file 1 [file nutrients-18-02515-s001.zip › Supplementary file S1.pdf]

Table S1. Participant's individual food and beverage consumption over the past 7 days reported using the Carotenoid Intake Screener (n = 278).

Food Item	Servings consumed (Mean ± SD)	Range of servings consumed
Apple (1 medium)	1.14 ± 1.62	0, 10
Avocado (½ cup, cubed)	1.21 ± 1.76	0, 10
Banana (1 medium)	1.69 ± 1.98	0, 12
Cantaloupe (1 slice, 1/8 of medium melon)	0.2 ± 1.04	0, 12
Fruit and Vegetable Smoothie or drink (12 fl. oz.)	0.88 ± 1.62	0, 12
Grapefruit (1 small)	0.31 ± 2.38	0, 33
Mango (½ mango)	0.37 ± 1.01	0, 7
Orange or Tangerine (1 medium)	1.17 ± 2.13	0, 15
Orange Juice (8 fl. oz.)	0.66 ± 1.38	0, 10
Watermelon (1 ½ cups)	0.35 ± 1.02	0, 6
Asparagus, cooked (6 medium spears)	0.29 ± 0.74	0, 4
Bell Peppers, cooked or raw (½ small or ¼ cup, chopped)	1.43 ± 1.66	0, 10
Broccoli, cooked or raw (1 cup)	1.18 ± 1.32	0, 6
Carrots, cooked or raw (1 cup)	1.38 ± 2.05	0, 20
Carrot Juice, 100% (8 fl. oz.)	0.06 ± 0.63	0, 10
Green beans, cooked (½ cup)	0.66 ± 1.08	0, 6
Greens (other than spinach)	1.04 ± 1.65	0, 10
Lettuce other than spinach	1.81 ± 2.28	0, 18
Spinach, cooked (1 cup)	0.56 ± 1.13	0, 8
Spinach, raw (1 cup)	0.79 ± 1.88	0, 18
Summer Squash [includes zucchini or spaghetti squash], cooked (1 cup, sliced)	0.40 ± 0.88	0, 5
Sweet Potato (1 cup)	0.43 ± 0.98	0, 8
Tomato, raw (¼ cup, chopped or sliced)	1.74 ± 2.34	0, 20
Tomato Juice or Vegetable Juice Cocktail, 100% (10 fl.	0.07 ± 0.41	0, 4
Tomato or Vegetable Soup (1 ½ cups)	0.35 ± 0.83	0, 4
Vegetable Combinations/Mixed Vegetables, cooked (1	1.23 ± 1.66	0, 10
Winter Squash [acorn, butternut, Hubbard, pumpkin], cooked (½ cup, cubes)	0.13 ± 0.63	0, 6
Pumpkin or Sweet Potato Pie (1 piece [⅛ of 9-inch	0.05 ± 0.28	0, 2
American Cheese (1 slice)	1.19 ± 1.72	0, 10
Burrito or Enchilada with Red Sauce (2 small)	0.36 ± 0.81	0, 6
Carrot Cake (1 slice [⅛ of 2-layer cake, 8–9" diameter])	0.03 ± 0.19	0, 2
Chili (1 cup)	0.40 ± 0.94	0, 6
Egg, cooked (1 large)	3.98 ± 4.60	0, 35
Fajita (1 cup)	0.24 ± 0.62	0, 4
Hamburger/Cheeseburger/Sandwich with Tomatoes or Tomato-based Sauce (1 sandwich)	0.73 ± 0.94	0, 5

Ketchup, Salsa, Barbecue Sauce, or Steak Sauce (1 tablespoon)	1.99 ± 2.43	0, 15
Lasagna, Ravioli, or Tortellini with Red Sauce (1 cup)	0.35 ± 0.75	0, 6
Meat with Vegetables and/or Tomato-based sauce (1 cup)	0.83 ± 1.30	0, 7
Pasta, Spaghetti, or Noodles with Red Sauce (1 cup)	0.95 ± 1.34	0, 11
Pizza with Red Sauce (2 slices [¼ of a large pizza])	1.03 ± 1.38	0, 12
Popcorn (3 cups, popped)	0.47 ± 0.93	0, 5
Spanish Rice (½ cup)	0.45 ± 1.21	0, 14
Taco, hard or soft (2 medium tacos)	0.61 ± 1.20	0, 12
Vegetable Chips (1 cup)	0.32 ± 0.88	0, 7
